# Acceptability of Covid-19 self-testing among Primary Health Care users

**DOI:** 10.11606/s1518-8787.2026060006399

**Published:** 2026-05-01

**Authors:** Fabiane Soares, Laio Magno, Thaís Regis Aranha Rossi, Suelen Seixas, Thiago Silva Torres, Débora Castanheira, Helena Lima, Ana Paula Pitanga, Valdiléa Gonçalves Veloso, Inês Dourado

**Affiliations:** I Universidade Federal da Bahia. Instituto de Saúde Coletiva. Salvador, BA, Brasil; II Universidade do Estado da Bahia. Departamento de Ciências da Vida. Salvador, BA, Brasil; III Fundação Oswaldo Cruz. Instituto Gonçalo Moniz. Salvador, BA, Brasil; IV Instituto Nacional de Infectologia Evandro Chagas. Fundação Oswaldo Cruz. Rio de Janeiro, RJ, Brasil; V Secretaria Municipal da Saúde de Salvador. Salvador, BA, Brasil

**Keywords:** Self-Testing, Covid-19 Testing, Primary Health Care

## Abstract

**OBJECTIVE:**

To identify factors associated with the acceptability of Covid-19 self-testing among socioeconomically vulnerable populations.

**METHODS:**

Cross-sectional study using data from the "TQT Covid-19" project, which involved users from 19 primary health care (PHC) units located in socioeconomically vulnerable areas in Salvador (BA) and Rio de Janeiro (RJ), Brazil. Data were collected between July 2022 and July 2023. Descriptive analysis of Covid-19 self-test acceptability was performed, and logistic regression models were used to estimate factors associated with acceptability, with respective 95% confidence intervals (95%CI).

**RESULTS:**

Among 7,939 study participants, 45.8% (95%CI 44.75–46.95) reported willingness to use a Covid-19 self-test. In the analysis of associated factors, regarding sociodemographic profile, non-Black individuals (OR_a_ = 1.17; 95%CI 1.02–1.34), cisgender men (OR_a_ = 1.23; 95%CI 1.12–1.37), and participants with higher educational levels (OR_a_ = 1.60; 95%CI 1.43–1.79) were more likely to accept the self-test. Those with prior knowledge of the self-test (OR_a_ = 2.33; 95%CI 2.11–2.58) and those previously diagnosed with Covid-19 (OR_a_ = 1.17; 95%CI 1.05–1.28) also reported higher acceptance.

**CONCLUSIONS:**

Provision of Covid-19 self-testing should be considered as a complement to testing within the public health system, especially due to its acceptance among vulnerable populations and the difficulties in accessing testing in many Brazilian regions. During periods of increased Covid-19 incidence, self-testing may serve as an important strategy for mass case detection, provided that access and knowledge are expanded so communities can play an active role in SARS-CoV-2 epidemiological surveillance.

## INTRODUCTION

Brazil reported its first case of Covid-19 on February 26, 2020^1^, and is among the South American countries most severely affected by the Covid-19 pandemic^2^. During the early stages of the pandemic, the healthcare system was overwhelmed by the demands for testing and care of individuals affected by the novel coronavirus^2,3^.

Mass vaccination strategies against Covid-19, along with diagnosis and epidemiological surveillance, combined with the training and empowerment of local communities, are essential for controlling the pandemic in low- and middle-income populations^4^. In this context, the public health system can leverage technological innovations, such as self-testing for SARS-CoV-2 antigen detection in asymptomatic and mildly symptomatic cases. This approach facilitates access to testing among vulnerable populations and those living in hard-to-reach areas, potentially contributing to a reduction in virus transmission among healthy individuals^2^.

The World Health Organization (WHO) has recognized the critical role of self-testing and has encouraged its use to complement existing laboratory testing, thereby expanding Covid-19 testing capacity in various countries^5^. Widespread testing remains essential for identifying both symptomatic and asymptomatic individuals, enabling timely clinical management and reducing further transmission^6^.

On January 28, 2022, the Brazilian National Health Surveillance Agency (*Agência Nacional de Vigilância Sanitária* – Anvisa) established regulations for the sale and use of Covid-19 self-tests in Brazil. The regulations specify that self-tests are intended for screening purposes, to enable and promote the earliest possible self-isolation of individuals with Covid-19. Sales of self-tests are permitted only through licensed pharmacies and healthcare establishments^7^.

Access to healthcare and Covid-19 testing in Brazil is heterogeneous and influenced by socioeconomic disparities, as well as by the availability and distribution of diagnostic services and equipment^8-12^. Screening within Primary Health Care (PHC) is one of the main public health measures recommended by the WHO for the early detection of individuals infected with SARS-CoV-2^5^. Although rapid testing performed by healthcare professionals is considered an accessible option^13^, its implementation in certain contexts and populations is limited by shortages of personnel, constraints within health services, and/or system overload during periods of increased demand, such as outbreaks or public health emergencies^11,14^.

In this context, the Covid-19 self-test is a safe health technology that can be readily made available to members of underserved and vulnerable communities, with the goal of expanding testing^15^. Its use can promote the adoption of measures to control new infections and facilitate isolation and/or quarantine when appropriate. Consequently, this tool plays a fundamental role in guiding individual decision-making and mitigating risks within communities^16^.

To improve access to Covid-19 testing in vulnerable populations, self-tests may represent a viable and timely solution^17^. In this context, they can complement testing within the framework of participatory epidemiological surveillance in the Brazilian Unified Health System (*Sistema Único de Saúde –* SUS). However, unlike the HIV self-test distribution strategy launched in December 2018^18^, the distribution of Covid-19 self-tests within SUS has not yet been implemented. Furthermore, there is limited information on the acceptability of Covid-19 self-tests among populations experiencing high social vulnerability, who could potentially benefit from this technology. This study aimed to identify factors associated with the acceptability of Covid-19 self-tests among residents of socioeconomically vulnerable areas in the municipalities of Salvador and Rio de Janeiro.

## METHODS

### Study Design

This cross-sectional study utilized data from the research titled "Expansion of testing, quarantine and telemonitoring strategies to contribute to the fight against the Covid-19 pandemic in Brazil" (TQT Covid-19 Study), conducted with users of 19 PHC units located in neighborhoods of high socioeconomic vulnerability in Salvador (BA) and Rio de Janeiro (RJ)^19^. These units provided Covid-19 testing to all individuals meeting epidemiological criteria, such as exhibiting symptoms consistent with Covid-19 or having close contact with a confirmed case, regardless of age.

Inclusion criteria for the TQT Covid-19 study were as follows: being a user of health units located in the Cabula/Beiru (Salvador) or Manguinhos (Rio de Janeiro) health districts; presenting symptoms suggestive of Covid-19 infection, with symptom onset between three and seven days prior to testing; or being a close contact of an individual diagnosed with Covid-19. For asymptomatic individuals, testing was indicated from the third to the seventh day after exposure. The exclusion criteria included the presence of active nasal bleeding, traumatic lesions, or other acute facial injuries, as well as the inability to provide informed consent.

Several communication and demand-generation strategies were implemented by the research team, Community Health Agents (CHAs), and PHC professionals to inform the community about testing at health units and to provide guidance on eligibility criteria. These strategies included community mobilization through printed project materials, such as posters and leaflets, as well as outreach via social media, community radio, and podcasts. Dissemination and testing activities were also conducted in schools, non-governmental organizations (NGO), religious institutions, and through the work of CHAs within the communities.

### Data Collection

The intervention was conducted in 19 PHC units, each over a six-month period, between July 2022 and July 2023. Of these, 17 were located in the Cabula/Beiru health district in Salvador, comprising 12 Family Health Units (*Unidades de Saúde da Família* – USF) and five Basic Health Units (*Unidades Básicas de Saúde* – UBS). Two USF were situated in the Manguinhos region of Rio de Janeiro.

All participants were registered on the study's data collection platform. Subsequently, before or after undergoing the rapid Covid-19 test, a socioeconomic and behavioral questionnaire was administered. The questionnaire included items on individual and family clinical history of comorbidities, clinical and laboratory history of Covid-19 infection, recent Covid-19–related symptoms, access to and use of health services, vaccination against SARS-CoV-2, and the acceptability of Covid-19 self-testing. Adolescents aged 12 to 17 completed the questionnaire themselves, while for children under 12, it was completed by their parents or guardians.

### Study Variables

- Outcome variable: acceptability of the self-test was assessed based on the following question: "Would you take a Covid-19 diagnostic test that you could administer yourself?" (yes; no);
- Other variables of interest: reasons for hypothetical refusal of a Covid-19 self-test (does not believe they can perform the test alone; prefers it to be performed by a professional; unsure what to do after receiving the result; swab collection is uncomfortable; does not want to perform it alone); source of prior knowledge about the self-test (internet; social media; friends; family; TV or newspaper; health services).

### Independent Variables

- Sociodemographic variables: city (Salvador; Rio de Janeiro); race/skin color (Black [Black and Brown]; non-Black [Asian, White, and Indigenous]); gender (cisgender woman, cisgender man, transgender woman, transgender man); age range (≤24 years, ≥ 25 to ≤ 54, ≥55 years); education level (no formal education; elementary school; high school; higher education); income (less than 2 minimum wages; more than 2 minimum wages);
- Covid-19–related variables: prior knowledge of Covid-19 self-testing (yes; no); previous Covid-19 swab testing (yes; no); prior Covid-19 diagnosis (yes; no); mask use for Covid-19 prevention (yes; no); access to health services (exclusively through SUS; access to private services); reported difficulty accessing Covid-19 testing (yes; no); experience of discrimination in health services (yes; no); perceived risk of Covid-19 infection (none to low risk; moderate to high risk).

### Data Analysis

A descriptive analysis of the study population and the acceptability of the self-test was conducted. Crude and adjusted logistic regression models were then estimated to identify factors associated with the acceptability of the Covid-19 self-test, with corresponding 95% confidence intervals (95%CI). Variables with a p-value < 0.20 in the bivariate analysis were included in the model, while those with p < 0.05 or deemed theoretically relevant were retained in the final model. Multicollinearity among the selected covariates was assessed using association tests, and the adequacy of the final models was evaluated with the Hosmer-Lemeshow goodness-of-fit test^20^, considering a significance level of 0.05.

### Ethical Issues

All individuals invited to participate received a verbal explanation of the study objectives, methodology, and protocol procedures. Written informed consent was obtained from all participants. Individuals aged 18 years or older signed an informed consent, while consent for participants under 18 years of age was obtained from their parents or legal guardians. Adolescents aged 12–17 years who agreed to participate also signed an assent form.

The study protocol was approved by the WHO Research Ethics Committee (ERC) (#CERC.0128A and #CERC.0128B) and by the local ethics committees in each participating city (Salvador, ISC/UFBA: #53844121.4.1001.5030; and Rio de Janeiro, INI/Fiocruz: #53844121.4.3001.5240, ENSP/Fiocruz: #53844121.4.3001.5240, and SMS/RJ: #53844121.4.3002.5279).

## RESULTS

A total of 7,939 participants completed the socio-behavioral questionnaire and were included in this analysis. The majority were from Salvador — Bahia (74.5%), identified as Black or Brown (84.3%), were cisgender women (69.6%), aged 25–54 years (61.3%), had completed high school education (56.4%), and reported a family income equal to or less than two minimum wages (≤ R$ 2,424.00) — 45.2%. Most participants indicated exclusive access to health services through the SUS (69.2%) and had prior experience with Covid-19 testing (73.2%), without difficulties in obtaining tests (91.1%) and without previous experiences of discrimination in health services (93.9%). Additionally, the majority reported a moderate or high perceived risk of Covid-19 infection (71.6%) and regular mask use (58.7%). Slightly more than one-third reported previous Covid-19 infection (39.3%), as presented in Table 1.

**Table 1:** Characteristics of participants enrolled in the study (n = 7,939). TQT Covid-19, Salvador and Rio de Janeiro.

Characteristics		n	%
**Sociodemographic**			
City			
	Salvador	5,913	74.50
	Rio de Janeiro	2,024	25.50
Race/skin color			
	Non-Black	1,218	15.72
	Black	6,528	84.27
Gender identity			
	Cisgender woman	5,356	69.58
	Cisgender man	2,330	30.27
	Transgender woman	9	0.12
	Transgender man	3	0.04
Age range (years)			
	≤ 24	1,321	16.64
	≥ 25 and < 55	4,867	61.30
	≥ 55	1,751	22.06
Education			
	No formal education	70	0.90
	Elementary school	1,915	24.75
	High school or incomplete higher education	4,364	56.40
	Complete higher education	1,388	17.94
Family income			
	≤ 2 minimum wages	3,591	45.23
	> 2 minimum wages	1,394	17.56
	Refused to answer	2,954	37.21
**Covid-19–related and healthcare access**			
Prior knowledge of self-test			
	Yes	4,786	60.28
	No	3,153	39.72
Access to health services			
	Exclusively through SUS	5,497	69.24
	Access to private health services	2,442	30.76
Previous Covid-19 testing			
	Yes	2,032	73.16
	No	5,539	26.84
Difficulty accessing Covid-19 testing			
	Yes	277	8.86
	No	2,851	91.14
Perceived risk of Covid-19 infection			
	None to low risk	2,255	28.42
	Moderate to high risk	5,679	71.58
Previous Covid-19 diagnosis			
	Yes	3,123	39.34
	No	4,816	60.66
Regular mask use			
	Yes	4,661	58.72
	No	3,277	41.28
Experience of discrimination in health services			
	Yes	484	6.10
	No	7,455	93.90

SUS: Unified Health System.

More than half of the participants reported prior knowledge of the Covid-19 self-test (60.3%). The main sources of information were television or newspapers (33.5%), healthcare professionals (18.7%), friends (16.7%), and social media (13.7%). Overall acceptability of self-testing was 45.8% (95%CI 44.8–46.9). Acceptability was analyzed across three periods of highest incidence during the data collection phase — July to October 2022; November to February 2023; and March to July 2023 — and no significant variation was observed (45.5, 46.4, and 43.9%, respectively). The main reasons cited for hypothetical refusal of the Covid-19 self-test were uncertainty about performing the test correctly (56.7%), preference to be tested by a healthcare professional (32.8%), lack of knowledge on what to do after a positive result (5.2%), and finding the oral swab uncomfortable (5.3%), as shown in Figure.

**Figure:**
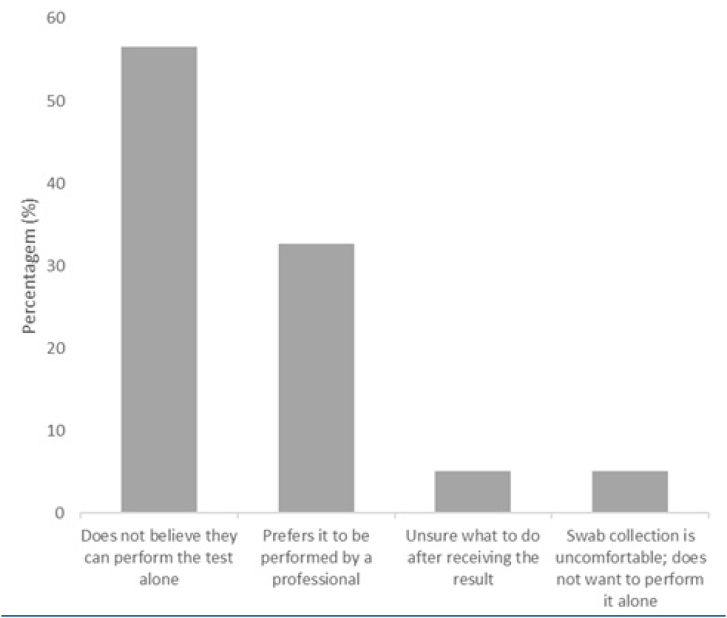
Reasons for hypothetical refusal to use a Covid-19 self-test. TQT Covid-19, Salvador and Rio de Janeiro.

In the multivariate analysis of sociodemographic variables, self-test acceptability was higher among participants who were predominantly White (OR_a_ = 1.17; 95%CI 1.02–1.34), cisgender men (OR_a_ 1.23; 95%CI 1.12–1.37), and those with higher education levels (OR_a_ = 1.60; 95%CI 1.43–1.79). Additionally, among variables related to SARS-CoV-2 infection, acceptability was higher among participants with prior knowledge of self-testing (OR_a_ = 2.33; 95%CI 2.11–2.58) and among those who reported previous SARS-CoV-2 infection before the study (OR_a_ = 1.16; 95%CI 1.06–1.28), compared with participants who were unaware of self-testing or had no prior infection, respectively (Table 2).

**Table 2:** Crude and adjusted logistic regression models of Covid-19 self-test acceptability and associated factors among PHC users. TQT Covid-19, Salvador and Rio de Janeiro.

		OR	95%CI	OR adjusted^a^	95%CI
City					
	Salvador	1			
	Rio de Janeiro	1.00	0.90–1.10		
Race/skin color					
	Black and Brown	1		1	
	Non-Black	1.29	1.14–1.46	1.17	1.02–1.34
Gender identity					
	Cisgender woman	1		1	
	Cisgender man	1.17	1.06–1.28	1.23	1.12–1.37
Education					
	No formal education to elementar school	1		1	
	Complete high school to complete higher education	1.86	1.67–2.07	1.60	1.43–1.79
Family income					
	≤ 2 minimum wages	1			
	> 2 minimum wages	1.56	1.38–1.76		
Prior knowledge of self-test					
	No	1			1
	Yes	2.37	2.16–2.59	2.33	2.11–2.58
Access to health services					
	Exclusively through SUS	1			
	Access to private health services	1.39	1.26–1.53		
Previous Covid-19 testing					
	No	1			
	Yes	1.30	1.18–1.45		
Difficulty accessing Covid-19 testing					
	No	1			
	Yes	1.18	0.92–1.51		
Perceived risk of Covid-19 infection					
	None to low risk	1			
	Moderate to high risk	1.11	1.00–1.22		
Previous Covid-19 diagnosis					
	No	1		1	
	Yes	1.31	1.20–1.44	1.16	1.05–1.28
Regular mask use					
	No	1			
	Yes	0.98	0.90–1.08		
Experience of discrimination in health services					
	No	1			
	Yes	1.25	1.05–1.51		

OR: odds ratio; 95%: 95% confidence interval; PHC: Primary Health Care.

a Adjusted for study site.

## DISCUSSION

Acceptability is a concept used to assess the introduction of a new health technology and varies according to individuals’ perceptions of the method or strategy, as well as individual, contextual, and social factors^21^. Studies conducted with adult populations in Brazil, Europe, Asia, and Africa have shown that more than half of participants expressed willingness to use the self-test^2,22-25^.

In the present study, less than half of the population indicated willingness to perform a Covid-19 self-test, suggesting that it is accepted by only a portion of individuals. Acceptability may increase when considering its availability through the SUS^22^. The literature indicates that the primary advantages of self-testing are the shorter time required and the faster results — compared with tests conducted in healthcare facilities^23^.

With the increase in case numbers, continued vigilance regarding SARS-CoV-2 infections is essential^26^. Covid-19 testing using molecular methods, such as reverse transcriptase polymerase chain reaction (RT-PCR), remains centralized and relatively inaccessible. Rapid antigen tests (Ag-RDTs) administered by healthcare professionals, although more accessible and adaptable for use in various settings^3^, are not available to the entire population. In this context, Covid-19 self-testing can help expand access to testing, thereby contributing to the control of virus transmission^2,17^.

Qualitative studies conducted in Peru and Indonesia have emphasized that self-testing should be easily accessible to those who could benefit most, particularly individuals with limited financial resources and/or those living in peripheral or rural areas. These studies also suggest that self-tests could be widely distributed through pharmacies, health supply stores, schools, markets, offices or factories, and neighborhood associations^16,27^.

The introduction of self-testing into SUS was recommended by the National Health Council in January 2020^28^. To overcome barriers to Covid-19 testing in certain contexts, the provision of self-testing by the public health system is essential, particularly for socioeconomically disadvantaged populations^27^. This perspective was also emphasized by potential implementers of self-testing programs and civil society representatives in a study conducted in Nigeria, with the aim of reducing inequities in access to Covid-19 testing^17^. In this context, offering self-tests within PHC services should be considered, with attention to their accessibility in communities with high socioeconomic vulnerability.

Self-testing is an important tool for expanding access to Covid-19 diagnosis, enabling individuals to quickly identify infection and implement measures to reduce virus transmission^29^, particularly among populations with limited access to healthcare services. Furthermore, widespread use of self-testing can facilitate the rapid identification of new outbreaks^30,31^. A key challenge lies in structuring the healthcare network to manage and accommodate the demand generated by users diagnosed through self-testing and to ensure appropriate follow-up.

Covid-19 self-testing has been implemented in several developed countries, demonstrating reliability, feasibility, and high acceptability among the population^31,32^. Remaining challenges include linking individuals who test positive to healthcare services, particularly in relation to stigma and the difficulties of self-isolation due to financial losses incurred during isolation^33^.

Furthermore, challenges to the large-scale implementation of self-testing still exist, such as high cost, unequal access, distrust regarding the accuracy and reliability of the test, difficulty in performing it according to the instructions in the package insert, and the correct interpretation of the results^30^. These findings highlight the importance of efficiently structuring the provision and implementation of self-testing in the public network. To this end, it is essential to promote access to self-testing among vulnerable populations, which requires a broad communication and community engagement strategy so that they have full knowledge of the method and how to use it^29^. In this sense, it is important to consider successful experiences of support on online platforms that can guide users, clarify doubts about the test, facilitate case notification, contact tracing, and referrals to the health care network when necessary^30,32,34-36^.

In the event of Covid-19 outbreaks, self-testing can serve as an accessible screening tool for socioeconomically vulnerable populations, facilitating mass case detection, particularly given that SUS health services often become overloaded^2^. In this study, more than half of respondents reported prior awareness of Covid-19 self-testing, with newspapers and television being key sources of information, alongside social media and healthcare professionals.

Among the reasons for refusal, the most common was participants’ perception that they would be unable to perform the test independently, reflecting apprehension, difficulty, lack of skill, or unfamiliarity with this type of technology. This underscores the need to increase accessibility of SARS-CoV-2 self-tests in communities and to raise awareness of their availability through various communication channels — including television and digital media campaigns — while providing guidance on proper use and steps to follow in the event of a positive result. Furthermore, once self-tests are available in primary health care, community health workers can serve as valuable allies in distributing the tests and instructing the community on their use.

In the studied population, higher acceptability of the self-test was observed among cisgender men, non-Black individuals, those with higher education levels, and participants who had previously had Covid-19 or were already familiar with the method. It can be hypothesized that the higher acceptance among men may be related to greater confidence in performing the test independently. Furthermore, in Brazil, women tend to be more attentive to health care and utilize health services more frequently than men^37^. In this context, men may be more interested in self-testing because it eliminates the need to visit health units, whereas women may prefer testing in the presence of a healthcare professional, as suggested by a study conducted in Indonesia^25^.

Supporting the present study, surveys conducted with adults in Europe, Asia, and Africa have shown that individuals with higher education levels were more likely to accept Covid-19 self-testing^22,25,38^. A similar pattern was observed in a population-based survey in São Paulo (Brazil), where the most educated respondents who perceived themselves to be at high risk of Covid-19 infection were more accepting of self-testing, even if they had to pay for it^2^. It can be inferred that more educated individuals are more willing to perform self-testing because they feel confident in their technical and cognitive ability to follow the instructions provided, whether through the package insert or other audiovisual resources, without the assistance of a healthcare professional. In this context, in Brazil, the White population is generally more socioeconomically advantaged^39^, with higher education levels and greater access to health technologies^40^, and therefore may be more inclined to use self-tests due to feeling capable and prepared to test themselves independently.

Participants who reported prior knowledge of the Covid-19 self-test also expressed greater interest in the method. Adequate access to and understanding of testing can influence individuals’ perceptions of self-testing, increasing awareness that it is a safe diagnostic tool and a reliable alternative to conventional testing^27^. The self-test was recommended by Anvisa in early 2022^7^, suggesting that individuals familiar with it were likely aware that it is a validated, safe diagnostic method endorsed by the relevant health authorities, which may have enhanced their confidence in using it. It is important to note that respondents were surveyed in health units where they were scheduled to undergo Covid-19 testing; that is, they belonged to communities served by PHC services and were able to access these services. Therefore, participants in this study may have had greater access to Covid-19 information and fewer barriers to testing compared with other population groups.

Participants who had previously experienced Covid-19 were also more willing to perform the self-test. This may be related to the importance they attributed to obtaining a diagnosis — enabling timely care and preventive measures — and their recognition of the self-test as a convenient alternative for confirming infection.

Regarding the use of self-tests, another challenge is determining how to proceed in the event of a positive result. Recent studies have reported that most users (approximately 90%) would inform their close contacts of a positive result and initiate isolation, with these contacts seeking post-test counseling at health units^2,22,23,25^. Therefore, in addition to ensuring accessibility and dissemination of self-tests within communities, it is important to establish a communication channel, such as software or a website, through which users can easily report test results. This facilitates linkage to the healthcare network for care and follow-up^16^, including case monitoring and contact tracing.

In the event of future Covid-19 outbreaks, self-testing should be considered as a complementary tool within the Brazilian public health system, enabling users to act as active participants in the epidemiological surveillance of SARS-CoV-2 in their communities. When integrating self-testing into public healthcare services, its provision should account for the socioeconomic and demographic characteristics of the population and be supported by clear and accessible communication strategies to ensure that a broad segment of the population can benefit from and utilize the method.

## Data Availability

The datasets are available from the corresponding author upon request.
