# Supplementary material for: Acceptability of Covid-19 self-testing among Primary Health Care users
Source: Rev Saude Publica. 2026 May 1;60(Suppl 1):e10s. doi: 10.11606/s1518-8787.2026060006399 (PMC13138127; doi:10.11606/s1518-8787.2026060006399)
Supplement: Translation

# Translation: Aceitabilidade do autoteste para Covid-19 entre usuários da Atenção Primária à Saúde

**Authors:** Fabiane Soares, Laio Magno, Thaís Regis Aranha Rossi, Suelen Seixas, Thiago Silva Torres, Débora Castanheira, Helena Lima, Ana Paula Pitanga, Valdiléa Gonçalves Veloso, Inês Dourado

**Affiliations:** I Universidade Federal da Bahia. Instituto de Saúde Coletiva. Salvador, BA, Brasil; II Universidade do Estado da Bahia. Departamento de Ciências da Vida. Salvador, BA, Brasil; III Fundação Oswaldo Cruz. Instituto Gonçalo Moniz. Salvador, BA, Brasil; IV Instituto Nacional de Infectologia Evandro Chagas. Fundação Oswaldo Cruz. Rio de Janeiro, RJ, Brasil; V Secretaria Municipal da Saúde de Salvador. Salvador, BA, Brasil

**Keywords:** Autoteste, Teste para Covid-19, Atenção Primária à Saúde

## Abstract

**OBJETIVO:**

Identificar os fatores associados à aceitabilidade do autoteste para Covid-19 entre pessoas em situação de vulnerabilidade socioeconômica.

**MÉTODOS:**

Estudo transversal com dados do "TQT Covid-19", que envolveu usuários de 19 unidades da Atenção Primária à Saúde (APS), localizadas em áreas de alta vulnerabilidade socioeconômica em Salvador (BA) e no Rio de Janeiro (RJ). A coleta de dados ocorreu entre julho de 2022 e julho de 2023. Foi realizada a análise descritiva da aceitabilidade de autoteste de Covid-19, bem como o uso de regressão logística para estimar fatores associados à aceitabilidade do autoteste para Covid-19 e os respectivos intervalos de confiança a 95% (IC95%).

**RESULTADOS:**

Dos 7.939 participantes do estudo, 45,8% (IC95% 44,75–46,95) afirmaram estar dispostos a utilizar o autoteste de Covid-19. Na análise de fatores associados, em relação ao perfil sociodemográfico, pessoas não negras (OR_a_ = 1,17; IC95% 1,02–1,34), homens cisgênero (OR_a_ = 1,23; IC95% 1,12–1,37) e indivíduos com maior nível de escolaridade (OR_a_ = 1,60; IC95% 1,43–1,79) foram os que mais aceitaram o autoteste. Aqueles que já tinham conhecimento sobre o autoteste (OR_a_ = 2,33; IC95% 2,11–2,58) e os que haviam sido diagnosticados anteriormente (OR_a_ = 1,17; IC95% 1,05–1,28) relataram uma maior aceitação do método.

**CONCLUSÕES:**

É importante considerar a disponibilização do autoteste para Covid-19 como complemento à testagem no sistema de saúde público, especialmente devido à sua aceitação entre populações vulneráveis e às dificuldades de acesso à testagem em muitas localidades brasileiras. Em situações de aumento da incidência de Covid-19, o autoteste pode ser uma estratégia importante para a detecção em massa de novos casos, desde que ampliados o acesso e o conhecimento sobre ele, de modo que as comunidades possam desempenhar um papel ativo na vigilância epidemiológica do SARS-CoV-2.

## INTRODUÇÃO

O Brasil registrou seu primeiro caso de Covid-19 em 26 de fevereiro de 2020^1^ e está entre os países da América do Sul onde a pandemia de Covid-19 atingiu mais duramente a população^2^. Nos anos iniciais da pandemia, o sistema de saúde esteve sobrecarregado por atender às demandas de testagem e de cuidado da população afetada pelo novo coronavírus^2,3^.

As estratégias de vacinação em massa contra a Covid-19, o diagnóstico e a vigilância epidemiológica, combinados com a capacitação e a instrumentalização de comunidades locais, são necessários para controlar a pandemia em populações de baixa e média renda^4^. Nesse contexto, o sistema de saúde público pode lançar mão de inovações tecnológicas, como autoteste para detecção de antígeno do SARS-CoV-2 em casos assintomáticos e de sintomas leves, facilitando o acesso à testagem entre grupos mais vulneráveis e/ou que vivem em áreas de difícil acesso, o que pode contribuir para diminuição da transmissão do vírus entre indivíduos saudáveis^2^.

A Organização Mundial da Saúde (OMS) reconheceu o papel fundamental dos autotestes e incentivou o seu uso para complementar os testes laboratoriais atuais com o propósito de ampliar a capacidade de testagem para Covid-19 nos países^5^. A testagem generalizada continua sendo de suma importância para identificar indivíduos sintomáticos e assintomáticos, facilitando o manejo clínico oportuno e reduzindo a transmissão posterior^6^.

A Agência Nacional de Vigilância Sanitária (Anvisa) regulamentou, no dia 28 de janeiro de 2022, os requisitos para comercialização e utilização de autotestes para Covid-19 no Brasil. Foi regulamentado que o autoteste deve ser utilizado para triagem, de modo a permitir e promover o autoisolamento mais precocemente possível de pessoas com Covid-19. A comercialização do autoteste no país é permitida apenas em farmácias e estabelecimentos de saúde licenciados^7^.

O acesso aos cuidados em saúde e à testagem de Covid-19 no país é variado e influenciado por disparidades socioeconômicas, bem como pela oferta e distribuição de serviços e equipamentos de diagnóstico^8-12^. A triagem no âmbito da Atenção Primária à Saúde (APS) é uma das principais ações de saúde pública recomendadas pela OMS para a detecção precoce de indivíduos infectados pelo SARS-CoV-2^5^. Embora a testagem rápida realizada por profissionais de saúde seja considerada uma opção acessível^13^, sua aplicação em determinados contextos e populações é restrita devido à escassez de profissionais, a limitações nos serviços de saúde e/ou à sobrecarga do sistema diante do aumento da demanda em situações de surtos ou emergências de saúde pública^11,14^.

Nesse sentido, o autoteste para Covid-19 é uma tecnologia de saúde segura, que pode ser disponibilizada facilmente entre membros de comunidades periféricas e vulneráveis com o objetivo de ampliação da testagem^15^. A partir de seu uso, o autoteste pode estimular a adoção de medidas de controle de novas infecções e possibilitar a realização de isolamento e/ou quarentena, quando oportuno. Assim, essa ferramenta desempenha um papel fundamental na orientação de decisões individuais e na mitigação de riscos em comunidades^16^.

Para facilitar o acesso à testagem de Covid-19 em populações vulneráveis, os autotestes podem representar uma solução viável e oportuna^17^. Nesse sentido, eles podem complementar a testagem no contexto de uma vigilância epidemiológica participativa no Sistema Único de Saúde (SUS). Entretanto, ainda não foi implementada a sua distribuição no SUS, ao contrário do que ocorreu com a estratégia de distribuição de autotestes de HIV, lançada em dezembro de 2018^18^. Além disso, há pouca informação sobre a aceitabilidade do autoteste de Covid-19 entre populações em situação de alta vulnerabilidade social, que poderiam se beneficiar dessa tecnologia. Este artigo tem como objetivo identificar os fatores associados à aceitabilidade do autoteste para a Covid-19 entre residentes de áreas com vulnerabilidade socioeconômica nos municípios de Salvador e do Rio de Janeiro.

## MÉTODOS

### Desenho do Estudo

Estudo transversal com dados do estudo intitulado "Ampliação das estratégias de testagem, quarentena, saúde digital e telemonitoramento para o enfrentamento da pandemia de Covid-19 no Brasil" (Estudo TQT Covid-19), feito com usuários de 19 unidades da APS, localizadas em bairros com alta vulnerabilidade socioeconômica em Salvador (BA) e no Rio de Janeiro (RJ)^19^. Nessas unidades foram ofertadas testagem para Covid-19 a todas as pessoas que tivessem indicação segundo critérios epidemiológicos, tais como apresentar sintomas compatíveis com Covid-19 ou contato próximo com um caso confirmado, independentemente da idade.

Os critérios de inclusão no estudo TQT Covid-19 foram: ser usuário de unidades de saúde situadas nas comunidades do distrito sanitário do Cabula/Beiru (Salvador) e de Manguinhos (Rio de Janeiro); estar com sintomas sugestivos de infecção por Covid-19, com o início dos sintomas entre três a sete dias do momento da realização da testagem, ou ser contactante próximo de pessoa diagnosticada com Covid-19. Se assintomático, a testagem era indicada após o terceiro e até o sétimo dia do contato. Os critérios de exclusão foram: apresentar sangramento nasal ativo ou lesões traumáticas ou outras lesões faciais agudas; e não ter capacidade de fornecer o consentimento esclarecido.

Diversas estratégias de comunicação e criação de demanda foram desenvolvidas pela equipe de pesquisa, por Agentes Comunitários de Saúde (ACS) e por profissionais de saúde da APS para informar a comunidade sobre a testagem nas unidades de saúde e orientar quanto aos critérios para a realização dos testes. Entre essas estratégias, houve mobilização da comunidade por meio de materiais impressos de divulgação e comunicação do projeto impressos, como cartazes e folhetos, além de ações em redes sociais, rádios comunitárias e *podcasts*. Também foram realizadas atividades de divulgação e testagem em escolas, organizações não governamentais, instituições religiosas e por meio da atuação dos ACS nos territórios.

### Coleta dos Dados

A intervenção ocorreu em 19 unidades de saúde da APS, com duração de seis meses em cada uma, entre julho de 2022 e julho de 2023. Dessas, 17 estavam localizadas no distrito sanitário Cabula/Beiru, em Salvador, sendo 12 unidades de saúde com a estratégia saúde da família (USF) e cinco unidades básicas de saúde (UBS). Duas USF localizavam-se no Rio de Janeiro, na região de Manguinhos.

Todos os participantes fizeram um cadastro na plataforma de coleta de dados do estudo. Posteriormente, antes ou após a realização do teste rápido para Covid-19, foi aplicado um questionário socioeconômico e comportamental, contendo questões referentes ao histórico clínico individual e familiar de comorbidades, ao histórico clínico e laboratorial de infecção por Covid-19, à sintomatologia recente de Covid-19, ao acesso e uso de serviços de saúde, à vacinação contra o SARS-CoV-2 e à aceitabilidade de autoteste para Covid-19. Os adolescentes entre 12 e 17 anos responderam ao questionário da pesquisa, enquanto os referentes aos menores de 12 anos foram respondidos pelos pais ou responsáveis.

### Variáveis do Estudo

- Variável de desfecho: a aceitabilidade do autoteste foi avaliada com base na seguinte pergunta: "Você faria um teste para diagnóstico da Covid-19 que você aplicasse em você mesmo?" (sim; não);
- Outras variáveis de interesse: razões para recusa hipotética de um autoteste para Covid-19 (não acredita que possa realizar o teste sozinho; prefere que seja feito por um profissional; não saberia o que fazer após o diagnóstico; a coleta de *swab* é desconfortável; não deseja fazer sozinho); fonte de conhecimento prévio acerca do autoteste (*internet*; mídias sociais; amigos; familiares; TV ou jornal; serviço de saúde).

### Variáveis Independentes

- Sociodemográficas: cidade (Salvador; Rio de Janeiro); raça/cor (negra [preta e parda]; não negra [asiática, branca e indígena]); gênero (mulher cisgênero, homem cisgênero, mulher transgênero/travestis, homem transgênero); grupo etário (≤ 24 anos, ≥ 25 a ≤ 54, ≥ 55 anos); escolaridade (sem formação formal; ensino fundamental; ensino médio; ensino superior); renda (menos de 2 salários-mínimos, mais de 2 salários-mínimos);
- Relacionados à Covid-19: conhecimento prévio sobre autoteste para Covid-19 (sim; não); realização prévia de teste de Covid-19 por *swab* (sim; não); diagnóstico prévio de Covid-19 (sim; não); uso de máscara para prevenção à Covid-19 (sim; não); acesso aos serviços de saúde (exclusivamente pelo SUS; tem acesso a serviços privados); relata dificuldade de acesso ao teste de Covid-19 (sim; não); experiência de discriminação nos serviços de saúde (sim; não); percepção de risco de se infectar pela Covid-19 (nenhum a baixo risco; moderado a alto risco).

### Análise dos Dados

Foi realizada uma análise descritiva da população do estudo e da aceitabilidade do autoteste e foram estimados modelos de regressão logística brutos e ajustados para os fatores associados à aceitabilidade do autoteste de Covid-19, com respectivos intervalos de confiança a 95% (IC95%). As variáveis com valor de p < 0,20 na análise bivariada foram inseridas no modelo, sendo mantidas no modelo final aquelas que apresentaram significância com p < 0,05 ou que foram consideradas relevantes devido à relevância teórica. A multicolinearidade entre as covariáveis selecionadas foi avaliada por testes de associação, e a adequação dos modelos finais foi verificada por meio do teste de ajuste de Hosmer-Lemeshow^20^, considerando um valor de p de 0,05.

### Questões Éticas

Todos os indivíduos convidados a participar receberam uma explicação verbal sobre os objetivos do estudo, a metodologia e os procedimentos do protocolo. Foi obtido o consentimento escrito de todos os participantes. Os maiores de 18 anos assinaram um termo de consentimento livre e esclarecido, sendo obtido o consentimento dos pais ou responsáveis dos menores de 18 anos. Os adolescentes entre 12 e 17 anos que aceitaram participar do estudo assinaram um termo de assentimento livre e esclarecido.

O protocolo do estudo foi aprovado no Comitê de Ética em Pesquisa da OMS (ERC) (#CERC.0128A e #CERC.0128B) e no comitê local de cada cidade que desenvolveu o estudo (Salvador, ISC/UFBA: #53844121.4.1001.5030; e Rio de Janeiro, INI/Fiocruz: #53844121.4.3001.5240, ENSP/Fiocruz: #53844121.4.3001.5240 e SMS/RJ: #53844121.4.3002.5279).

## RESULTADOS

Um total de 7.939 participantes do estudo responderam ao questionário sociocomportamental e foram incluídos nessa análise. Desses, a maioria foi proveniente de Salvador — BA (74,5%), era preta ou parda (84,3%), mulher cisgênero (69,6%), com idade entre 25 e 54 anos (61,30%), com ensino médio completo (56,4%) e com renda média familiar igual ou inferior a dois salários-mínimos (≤ R$ 2.424,00) — 45,2%. A maior parte dos participantes também indicou acesso aos serviços de saúde exclusivamente pelo SUS (69,2%), e com experiência prévia de testagem para Covid-19 (73,2%), sem dificuldades para se testar (91,1%), e sem experiência prévia de discriminação nos serviços de saúde (93,9%). A maioria também relatou risco de infecção por Covid-19 moderado ou alto (71,6%) e indicou usar máscara regularmente (58,7%). Um pouco mais de um terço relatou infecção prévia por Covid-19 (39,3%), conforme Tabela 1.

**Tabela 1:** Características dos participantes inscritos no estudo (n = 7.939). TQT Covid-19, Salvador e Rio de Janeiro.

Variáveis		n	%
**Sociodemográficas**			
Cidade			
	Salvador	5.913	74,50
	Rio de Janeiro	2.024	25,50
Raça/cor da pele			
	Não negros	1.218	15,72
	Negros	6.528	84,27
Identidade de gênero			
	Mulher cisgênero	5.356	69,58
	Homem cisgênero	2.330	30,27
	Mulher transgênero	9	0,12
	Homem transgênero	3	0,04
Grupo etário (anos)			
	≤ 24	1.321	16,64
	≥ 25 e < 55	4.867	61,30
	≥ 55	1.751	22,06
Escolaridade			
	Sem educação formal	70	0,90
	Ensino fundamental	1.915	24,75
	Ensino médio a superior incompleto	4.364	56,40
	Ensino superior completo	1.388	17,94
Renda familiar			
	≤ 2 salários-mínimos	3.591	45,23
	> 2 salários-mínimos	1.394	17,56
	Recusa responder	2.954	37,21
**Relacionadas à Covid-19 e acesso aos serviços de saúde**			
Conhecimento anterior sobre autoteste			
	Sim	4.786	60,28
	Não	3.153	39,72
Acesso aos serviços de saúde			
	Exclusivamente pelo SUS	5.497	69,24
	Acessa serviços de saúde privados	2.442	30,76
Realizou teste de Covid-19 anterior			
	Sim	2.032	73,16
	Não	5.539	26,84
Dificuldade de acesso para a testagem de Covid-19			
	Sim	277	8,86
	Não	2.851	91,14
Percepção de risco de se infectar por Covid-19			
	Nenhum a baixo risco	2.255	28,42
	Moderado a alto risco	5.679	71,58
Teve diagnóstico anterior de Covid-19			
	Sim	3.123	39,34
	Não	4.816	60,66
Relata uso regular de máscara			
	Sim	4.661	58,72
	Não	3.277	41,28
Experiência de discriminação nos serviços de saúde			
	Sim	484	6,10
	Não	7.455	93,90

SUS: Sistema Único de Saúde.

Mais da metade dos participantes indicou ter conhecimento prévio sobre o autoteste de Covid-19 (60,3%). As principais fontes de informação foram TV ou jornal (33,5%), profissionais do serviço de saúde (18,7%), amigos (16,7%) e mídias sociais (13,7%). A aceitabilidade para uso do autoteste foi de 45,8% (IC95% 44,8–46,9). Foi analisada a aceitabilidade desse autoteste em três momentos de maior incidência no período em que houve a coleta de dados — julho a outubro de 2022; novembro a fevereiro de 2023; e março a julho de 2023 — e não foi observada mudança significativa na aceitabilidade (45,5, 46,4 e 43,9%, respectivamente). As razões para recusa hipotética do autoteste para Covid-19 foram: não saber se conseguiriam realizar o teste por conta própria (56,7%), preferir fazer com um profissional de saúde (32,8%), não saber o que fazer após o diagnóstico (5,2%) e considerar o *swab* oral desconfortável (5,3%), conforme a Figura.

**Figura:** As razões para a recusa hipotética de uso do autoteste para Covid-19. TQT Covid-19, Salvador e Rio de Janeiro.

Na análise multivariada, no conjunto de variáveis sociodemográficas, observou-se que a aceitabilidade de autoteste foi maior entre os participantes majoritariamente brancos (OR_a_ = 1,17; IC95% 1,02–1,34), homens cisgênero (OR_a_ = 1,23; IC95% 1,12–1,37) e aqueles com nível de educação superior (OR_a_ = 1,60; IC95% 1,43–1,79). Além disso, no conjunto de variáveis relacionadas à infecção pelo SARS-CoV2, a aceitabilidade do autoteste foi maior entre os participantes que tinham conhecimento prévio sobre o autoteste (OR_a_ = 2,33; IC95% 2,11–2,58) e que relataram terem tido infecção pelo SARS-CoV2 antes do estudo (OR_a_ = 1,16; IC95% 1,06–1,28), em comparação com aqueles que desconheciam o autoteste, e sem infecção prévia, respectivamente (Tabela 2).

**Tabela 2:** Modelos de regressão logística brutos e ajustados da aceitabilidade de autoteste de Covid-19 e fatores associados entre usuários da APS. TQT Covid-19, Salvador e Rio de Janeiro.

		OR	IC95%	OR ajustada^a^	IC95%
Cidade					
	Salvador	1			
	Rio de Janeiro	1,00	0,90–1,10		
Raça/cor da pele					
	Pretos e pardos	1		1	
	Não negros	1,29	1,14–1,46	1,17	1,02–1,34
Identidade de gênero					
	Mulher cisgênero	1		1	
	Homem cisgênero	1,17	1,06–1,28	1,23	1,12–1,37
Escolaridade					
	Sem educação formal a ensino fundamental	1		1	
	Ensino médio completo a superior completo	1,86	1,67–2,07	1,60	1,43–1,79
Renda familiar					
	≤ 2 salários-mínimos	1			
	> 2 salários-mínimos	1,56	1,38–1,76		
Conhecimento anterior sobre autoteste					
	Não	1			1
	Sim	2,37	2,16–2,59	2,33	2,11–2,58
Acesso aos serviços de saúde					
	Exclusivamente pelo SUS	1			
	Acessa serviços de saúde privados	1,39	1,26–1,53		
Realizou teste de Covid-19 anterior					
	Não	1			
	Sim	1,30	1,18–1,45		
Dificuldade de acesso para a testagem de Covid-19					
	Não	1			
	Sim	1,18	0,92–1,51		
Percepção de risco de se infectar por Covid-19					
	Nenhum a baixo risco	1			
	Moderado a alto risco	1,11	1,00–1,22		
Teve diagnóstico anterior de Covid-19					
	Não	1		1	
	Sim	1,31	1,20–1,44	1,16	1,05–1,28
Relata uso regular de máscara					
	Não	1			
	Sim	0,98	0,90–1,08		
Experiência de discriminação nos serviços de saúde					
	Não	1			
	Sim	1,25	1,05–1,51		

OR: *Odds ratio*; IC95%: intervalo de confiança de 95%; APS: Atenção Primária à Saúde.

a Modelo ajustado pelo sítio do estudo.

## DISCUSSÃO

A aceitabilidade é um conceito utilizado para analisar a introdução de uma nova tecnologia em saúde e varia de acordo com as percepções dos sujeitos frente ao novo método ou à nova estratégia, bem como por fatores individuais, contextuais e sociais^21^. Estudos com população adulta no Brasil, na Europa, na Ásia e na África têm revelado que mais da metade dos entrevistados estaria disposta a usar o autoteste^2,22-25^.

No presente estudo, menos da metade da população estudada faria um autoteste diagnóstico da Covid-19, indicando que ele é aceito apenas por uma parcela da população. Essa aceitabilidade pode ser maior ao considerar a sua oferta pelo SUS^22^. A literatura indica que as principais vantagens para o uso do autoteste têm sido o menor tempo para realização e o resultado mais rápido — quando comparado aos demais testes que são realizados em serviços de saúde^23^.

Com o aumento do número de casos, é preciso manter a vigilância das infecções pelo SARS-CoV2^26^. A testagem para Covid-19 por métodos moleculares, como a reação em cadeia da polimerase com transcriptase reversa (RT-PCR), continua centralizada e pouco acessível, e os testes rápidos de antígenos (Ag-RDTs) aplicados por profissionais, apesar de serem mais acessíveis e flexíveis para serem realizados em diferentes locais^3^, não estão disponíveis a toda a população. Nesse contexto, o autoteste para Covid-19 pode ajudar na expansão do acesso à testagem, de modo a contribuir para o controle da transmissão do vírus^2,17^.

Estudos qualitativos realizados no Peru e na Indonésia têm salientado que a oferta do autoteste precisa ser de fácil acesso àqueles que podem se beneficiar do método, sobretudo entre os que têm menos recursos financeiros e/ou que residem em regiões mais periféricas ou rurais. Como possibilidade, indicam também que os autotestes poderiam ser amplamente disponibilizados em farmácias, lojas de equipamentos e utensílios da atenção à saúde, em escolas, mercados, escritórios ou fábricas e em associações de bairro^16,27^.

A introdução do autoteste no SUS foi recomendada pelo Conselho Nacional de Saúde em janeiro de 2020^28^. Para enfrentar a barreira da inacessibilidade da testagem para Covid-19 em alguns contextos, a oferta de autoteste pelo sistema público de saúde não pode ser negligenciada, sobretudo para populações desfavorecidas socioeconomicamente^27^. Tal afirmativa foi também pontuada por potenciais implementadores de programa de autoteste e representantes da sociedade civil em estudo na Nigéria, tendo em vista a redução das iniquidades do acesso à testagem para Covid-19^17^. Nesse sentido, a oferta na APS pode ser considerada, atentando-se para sua capilaridade em comunidades de alta vulnerabilidade socioeconômica.

O autoteste é uma ferramenta importante para ampliar o acesso ao diagnóstico de Covid-19, de modo que as pessoas possam identificar rapidamente a infecção e promover medidas para reduzir a disseminação do vírus^29^, especialmente entre populações com dificuldades em acessar serviços de saúde. Além disso, o seu uso em larga escala pode ajudar na identificação rápida de novos surtos^30,31^. Para tanto, coloca-se como desafio a estruturação da rede para atender e acomodar a demanda de usuários diagnosticados a partir de um autoexame e garantir o acompanhamento necessário.

O autoteste de Covid-19 foi implementado em vários países desenvolvidos, demonstrando ser uma ferramenta confiável, viável e de alta aceitação na população^31,32^. Os desafios ainda existentes são a vinculação aos serviços por parte dos usuários que têm resultado reagente, especialmente relacionado ao estigma e às dificuldades de se realizar o isolamento devido às perdas financeiras ocasionadas com o isolamento^33^.

Além disso, ainda existem desafios para a implementação em larga escala do autoteste, como o alto custo, o acesso desigual, a desconfiança quanto à acurácia e confiabilidade do teste, a dificuldade em realizá-lo conforme as instruções da bula e a correta interpretação dos resultados^30^. Essas evidências destacam a importância de estruturar de forma eficiente a oferta e a implementação do autoteste na rede pública. Para isso, é essencial promover o acesso ao autoteste entre populações vulneráveis, o que exige uma ampla estratégia de comunicação e envolvimento da comunidade, para que tenham pleno conhecimento do método e de como utilizá-lo^29^. Nesse sentido, é importante considerar experiências bem-sucedidas de suportes em plataformas *online* que possam orientar os usuários, esclarecer dúvidas sobre o teste, facilitar a notificação de casos, o rastreamento de contactantes e os encaminhamentos à rede de atenção à saúde, quando necessário^30,32,34-36^.

Na ocorrência de surtos da Covid-19, o autoteste pode ser uma ferramenta de triagem acessível às populações mais vulneráveis socioeconomicamente, promovendo detecção de casos em massa, considerando que os serviços de saúde da rede do SUS tendem a ficar sobrecarregados^2^. Neste estudo, mais da metade dos entrevistados indicou já ter escutado sobre o autoteste para Covid-19, e a comunicação pelos jornais e televisão se mostrou bastante importante, assim como pelas mídias sociais e por meio dos profissionais de saúde.

Dentre as razões para recusa, a mais frequente foi a percepção dos sujeitos de que não conseguiriam executar o teste sozinhos, o que demonstra receio, dificuldade, falta de habilidade ou ausência de usabilidade desse tipo de tecnologia. Coloca-se em evidência, portanto, a necessidade de tornar o autoteste para detecção do SARS-CoV-2 mais acessível às comunidades, assim como informá-las também da existência do método por meio de diferentes veículos de comunicação — o que inclui campanhas na televisão e nas mídias digitais — e orientá-las quanto ao uso e a como proceder nos casos de diagnóstico positivo. Além disso, uma vez disponível na APS, os agentes comunitários podem ser importantes aliados para distribuição e orientação à comunidade quanto ao uso desses autotestes.

Na população estudada, observou-se que os homens cisgêneros, os que não eram negros, os mais escolarizados, assim como os que tiveram Covid-19 anteriormente e que já conheciam o método, aceitaram melhor o autoteste. É possível hipotetizar que a melhor aceitação pelos homens tenha sido por eles se sentirem mais seguros do que as mulheres para realizar o teste em si próprios. Além disso, no Brasil, as mulheres são mais preocupadas com cuidados à saúde e utilizam mais os serviços de atenção à saúde do que os homens^37^. Nesse sentido, é razoável presumir que os homens tenham mais interesse no uso de autoteste, pois assim não precisariam ir a unidades de saúde para realizar a testagem; por outro lado, as mulheres podem não dar tanto valor à realização do autoteste no domicílio e preferir realizar o teste na presença de um profissional de saúde, conforme foi sugerido em um estudo feito na Indonésia^25^.

Corroborando com o presente trabalho, inquéritos realizados com adultos na Europa, na Ásia e em África observaram que indivíduos com maiores níveis de escolaridade aceitaram mais o autoteste para Covid-19^22,25,38^. O mesmo foi observado no inquérito de base populacional realizado em São Paulo (Brasil), no qual os entrevistados mais escolarizados e que se percebiam em alto risco de infecção pela Covid-19 aceitaram melhor o autoteste, ainda que tivessem que pagar pelo método^2^. Pode-se presumir que as pessoas mais escolarizadas tenham maior disposição a realizar o autoteste por avaliarem que têm condições técnicas e cognitivas de executá-lo sozinhas (a partir das instruções da bula e/ou de outros recursos audiovisuais), sem a necessidade de um profissional de saúde. Ainda nessa linha, no Brasil, a população branca, em geral, é também mais favorecida socioeconomicamente^39^, tendo maiores níveis de escolaridade e acesso a tecnologias em saúde^40^, e, por isso, pode ter mais interesse em usar autotestes por sentir-se segura e preparada para se testar sem o acompanhamento de um profissional.

Os participantes que reportaram já saber sobre o autoteste de Covid-19 também indicaram maior interesse no método. O acesso e a compreensão adequados sobre a testagem podem moldar opiniões e percepções das pessoas sobre o autoteste, de modo a conscientizar as populações de que esse é um método de diagnóstico seguro e uma alternativa confiável para testagem^27^. O autoteste passou a ser indicado pela Anvisa no início de 2022^7^, sendo possível presumir que as pessoas que tinham conhecimento sobre ele tinham também ciência de que se trata de um método de diagnóstico seguro, validado e indicado pelos órgãos de saúde responsáveis, levando-as a dar credibilidade ao teste e a usá-lo. Importante salientar que os entrevistados foram inquiridos em unidades de saúde, nas quais estavam para realizar testagem de Covid-19, ou seja, trata-se de um público pertencente a um território coberto por um serviço da APS e que conseguiu acessar o serviço. Considerando isso, é importante salientar que os participantes deste estudo poderiam ter maior acesso à informação referente à Covid-19, bem como ter menos dificuldade de acessar a testagem em um serviço quando comparados a outros grupos populacionais.

Por sua vez, os participantes que tiveram Covid-19 previamente também estavam mais dispostos a realizar o autoteste, e isso pode estar relacionado à importância percebida por eles em realizar a testagem e ter o diagnóstico de Covid-19 — de modo a viabilizar as medidas de cuidado e prevenção necessárias — e entenderem que o autoteste seria uma alternativa para ter o diagnóstico.

Quanto ao uso deste autoteste, outro desafio que se coloca é como proceder na ocorrência de um resultado positivo. Estudos recentes revelaram que, na ocasião de um resultado positivo, segundo relatos, a maioria dos usuários (em torno de 90%) comunicaria o diagnóstico aos seus contatos próximos e iniciaria o isolamento, sendo requisitado por parte destes o aconselhamento pós-testagem em unidades de saúde^2,22,23,25^. Assim, é importante, além de tornar o autoteste acessível e divulgá-lo para as comunidades, que seja definido um canal de comunicação, como por um *software* ou *site*, para que o usuário possa informar o resultado do teste de forma simples, de modo a ser vinculado à rede de serviços para atenção e prestação dos cuidados^16^, o que inclui o monitoramento dos casos e o rastreio de seus contactantes.

Na possibilidade de futuros surtos de Covid-19, o autoteste deve ser considerado para complementar a testagem no sistema de saúde público brasileiro, tornando os usuários agentes na vigilância epidemiológica do SARS-CoV-2 em suas comunidades. Mediante a integração do autoteste na prestação de cuidados da rede pública, a oferta deve levar em consideração as condições socioeconômicas e demográficas da população, sendo divulgada a partir de estratégias de comunicação claras e acessíveis, de modo que uma parcela da população possa se beneficiar e fazer uso do método.

## Data Availability

Os dados podem ser disponibilizados mediante solicitação ao autor correspondente.
